# *Staphylococcus caprae* bacteraemia and native bone infection complicated by therapeutic failure and elevated MIC: a case report

**DOI:** 10.1099/jmmcr.0.005112

**Published:** 2017-09-18

**Authors:** Carolyn A. Hilliard, Jad El Masri, Michihiko Goto

**Affiliations:** Department of Internal Medicine, University of Iowa Carver College of Medicine, 200 Hawkins Drive, Iowa City, IA 52242, USA

**Keywords:** *Staphylococcus caprae*, therapeutic failure, elevated MIC, bacteraemia, native joint infection, vancomycin

## Abstract

**Introduction.** *Staphylococcus caprae* is a coagulase-negative staphylococcus that has been reported in several cases as a human pathogen. However, it has rarely been reported as pathogen in native bone. Furthermore, the reported MIC levels noted in the literature for vancomycin were <2 µg ml^−^^1^making vancomycin a first line choice for infected patients.

**Case presentation.** We report a case of *Staphylococcus caprae* causing osteomyelitis of the lumbar spine and bacteraemia and resulting in sepsis and ultimately the demise of a patient despite appropriate prolonged antibiotic therapy.

**Conclusion.** *Staphylococcus caprae* has been reported as a human pathogen since 1983 when it was discovered. We report a case involving native bone infection which is rare in the absence of mechanical hardware. Furthermore, this strain had an elevated MIC for vancomycin which has not been reported in the literature.

## Abbreviations

MALDI-TOF, matrix-assisted laser desorption/ionization time-of-flight; MRI, magnetic resonance imaging.

## Introduction

*Staphylococcus caprae* is a coagulase-negative staphylococcus that was first described in 1983 after being isolated from goats where it causes mastitis [1–4]. *S. caprae* typically colonizes human skin, nose and nails and since 1983 it has been reported in several cases as a human pathogen causing peritonitis, meningitis, urinary tract infections, endocarditis, endophthalmitis, prosthetic joint infections, recurrent sepsis, bacteraemia and osteomyelitis [4–9]. Many risk factors for *S. caprae* have begun to emerge and include immunosuppression, diabetes, chronic renal failure, obesity, open or traumatic fractures and contact with sheep or goats [4, 6]. Importantly, several strains of *S. caprae* have been noted to produce toxic shock syndrome toxin and carry the gene *mecA* important for methicillin resistance, and have been noted to form a biofilm *in vitro* on prostheses or bone thought to be due to the presence of the gene *altC* in combination with the *ica* operon [1, 4, 6, 10]. Very few cases of *S. caprae* bone and joint infection occur in native bone and joints without prostheses, and there are few cases of bacteraemia not associated with line infections. In this case report we present *Staphylococcus caprae* infection of native vertebrae with resulting bacteraemia and ultimately failure of therapy, and summarize the current case reports of infection with this organism.

## Case report

Our patient was a 69-year-old man with a history of cryptogenic liver cirrhosis (diagnosed 3 months prior to this hospitalization), diabetes mellitus and a history of lumbar spine hemi-laminotomy in 2007. He was admitted to our institution after leaving an outside hospital emergency department for acute worsening of chronic lower back pain, and difficulty ambulating. The patient had been previously diagnosed with L4–L5 discitis with methicillin-susceptible *Staphylococcus caprae* bacteraemia 3 months prior to admission (drawn at outside hospital, MicroScan panels confirming sensitivity to vancomycin with MIC of <1 µg ml^−1^) that was treated for 9 weeks total with parenteral antibiotic therapy (initially ertapenem, followed by vancomycin for 6 weeks) at an outside institution. Despite antibiotic therapy, the patient continued to have low back pain, worsening subjective weakness and numbness in his legs and 2 weeks after finishing antibiotics he was unable to walk even with a walker due to pain. Prior to admission, he was evaluated by a neurosurgeon for his chronic back pain in clinic and was thought to have degenerative changes of the spine that were not amenable to surgery.

**Fig. 1.**
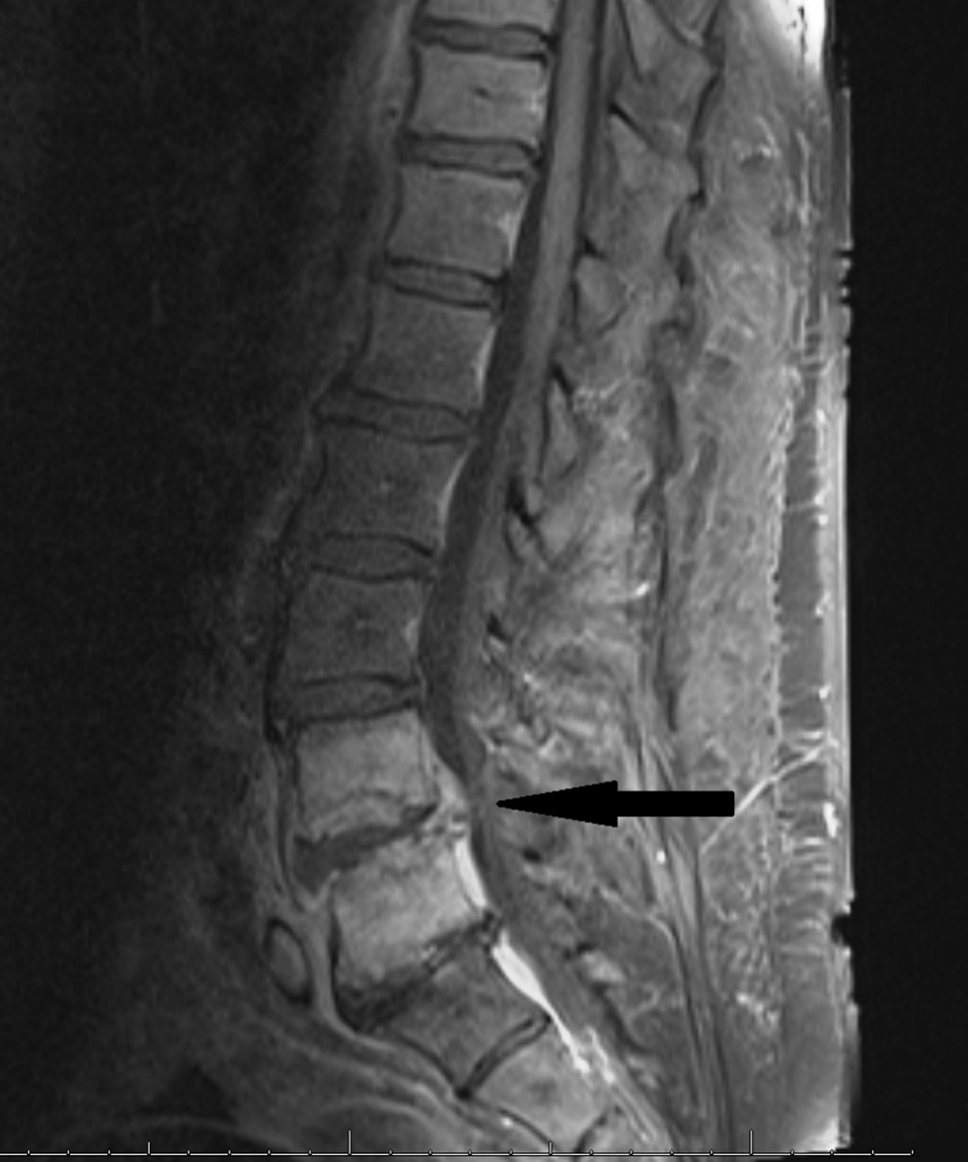
Lumbar spine MRI (T1 sequence with gadolinium enhancement). Arrow indicates L4–5 discitis/osteomyelitis with epidural extension.

The patient reported that the pain was in the lower back, radiating to the lower extremities, chronic with acute worsening over the 5 days preceding the evaluation. On examination, the patient had no fevers and had normal vital signs. He did not have point tenderness on his back, and had 2/5 strength documented in all his extremities, normal sensation and no documentation of gait. Laboratory tests revealed a normal white blood cell count of 9.2 K per mm^3^, sedimentation rate of 83 mm h^−1^, and C-reactive protein of 2.5 mg dl^−1^, with evidence of an acute kidney injury (serum creatinine level: 2.1 mg dl^−1^ with a normal baseline) and decompensated liver cirrhosis. Magnetic resonance imaging (MRI) of the lumbar spine revealed spinal discitis/osteomyelitis at L4–L5 level with epidural phlegmon resulting in severe canal stenosis at L4–L5 level (Fig. 1). Initially, antibiotics were held as the patient was still haemodynamically stable and a biopsy of affected spine lesion with cultures was desired to establish a microbiological diagnosis.

The patient rapidly developed progressive hepatic failure with severe coagulopathy and acute kidney injury after hospital admission. On hospital day 4, he developed gross haemoptysis with increasing oxygen requirements and was transferred to the medical intensive care unit. Chest x-ray revealed diffuse patchy opacities bilaterally, that were not present on admission. The patient was intubated and a bronchoscopy was performed showing evidence of diffuse alveolar haemorrhage. Blood cultures drawn at the outside hospital emergency department immediately prior to admission were growing *Staphylococcus caprae* with intermediate resistance to vancomycin documented in the report sent to our hospital (established by Vitek 2 at outside hospital, confirmed by manual MIC testing, although MIC result was 4 µg ml^−1^ per verbal report). The patient went into shock and was started on vasopressors. He was started on cefazolin for coverage of *Staphylococcus caprae* at the recommendation of our Infectious Disease specialists, in addition to vancomycin, piperacillin-tazobactam and doxycycline for additional coverage for possible infectious aetiologies for diffuse alveolar haemorrhage. Despite aggressive antimicrobial therapy and supportive care, the patient's haemodynamic status continued to deteriorate and the patient expired on hospital day nine.

Autopsy revealed necrotic and haemorrhagic L4–L5 vertebral bodies and disc spaces, with evidence of bilateral pleural effusions with diffuse alveolar haemorrhage. Tissue cultures from the affected vertebral bodies grew *Staphylococcus caprae* with MIC of 4 µg ml^−1^ for vancomycin established with automated microdilution method (Vitek 2; bioMérieux) and confirmed with Etest (bioMérieux).

## Discussion

*Staphylococcus caprae* has been noted as a human pathogen since the late 1980s after its discovery in 1983. There are reports in the literature of a variety of infections caused by *S. caprae* but by far the largest number of infections occur in bone and joint, and of these, the vast majority are in patients with orthopaedic devices in place. *S. caprae* has been uncommonly reported as a pathogen of native bone and here we report a case of native bone infection as the source of bacteraemia. No prior studies have shown a MIC of 4 µg ml^−1^ for vancomycin for *S. caprae;* prior studies publishing MICs are limited but were <2 µg ml^−1^ and recommended vancomycin as first line therapy against *S. caprae* [10]. At our institution, doxycycline was a viable alternative and cefazolin was chosen due to the consistent susceptibility of this individual’s *S. caprae* isolates to oxacillin (cephalosporins are not routinely tested when organism is a species of the genus *Staphylococcus*). Table 1 lists the three separate cultures of *S. caprae* during this individual’s clinical course as well as their MIC values. Interestingly, vancomycin began as MIC of 1 µg ml^−^^1^and then further MIC values were 4 µg ml^−^^1^.

**Table 1.** Antibiotics tested and their MIC (µg ml^−^^1^) values from blood cultures at two outside hospitals and from lumbar spine samples taken at autopsy

Outside hospital March 2016 (initial, blood):	Outside hospital July 2016 (blood):	Our institution July 2016 (lumbar spine):
Levofloxacin <0.5	Levofloxacin 0.5	
Clindamycin <0.25	Clindamycin 0.25	
Erythromycin <0.25	Erythromycin 1	Erythromycin 0.5
Oxacillin <0.25	Oxacillin <0.25	Oxacillin <0.25
Tetracycline <1	Tetracycline <1	Tetracycline <1
Bactrim <0.5	Bactrim <10	Bactrim <0.5
Vancomycin 1	Vancomycin Int (verbal =4)	Vancomycin 4
Penicillin >10 R		Rifampin <0.5
Ciprofloxacin <1		Doxycycline <0.5
Meropenum <2		
Gentamicin <1		

Several case reports of infections related to *S. caprae* are available in the literature. Table 2 lists most of the available case reports. There was also a case report that mentioned a 72-year-old Japanese man with recurrent *S. caprae* sepsis but this report was only available in Japanese [5]. A different study out of Japan did identify *S. caprae* in the urine of patients who had received chemotherapy but it was not evident whether it was infectious or not. However, in that report they did note a high incidence of methicillin resistance (11 % of all methicillin-resistant organisms were *S. caprae*), and that *S. caprae* totalled 6 % of all coagulase-negative staphylococcal species isolated^8^]. In 2004, Ross and colleagues published a study in which 10 of 36 neonates in their neonatal intensive care unit were colonized with *S. caprae*, of which six cases were bacteraemic, one case was a cerebrospinal fluid (CSF) shunt infection and onewas a vascular catheter-associated infection, and it was noted that 13 % of the *S. caprae* strains recorded carried the *mecA* gene [3].

**Table 2.** Summary of case reports involving *S. caprae* infections

Author and year of case(s)	Publication type	Site of infection	Clinical history
Vandenesch *et al.* 1988–1992 [9]	Case series report	UTI	46-year-old s/p medullary decompression for rhabdomyosarcoma 8-year-old previously healthy female
		Endocarditis	45-year-old previously healthy female, required mitral valve removal
		Bacteremia	67-year-old s/p ovariectomy for ovarian cancer, from her Port-a-Cath Neonate with umbilical catheter and coarctation of the aorta
Shuttleworth *et al.* 1990–1996 [10]	Case series report	Bone and joint infection	9 cases, 7 of which were traumatic fractures
		Toenail infection	1 case
		Bilateral otitis media	1 case
		Transplant patients	3 cases
Kanda *et al.* 1998–2000 [13]	Case series report	Bacterial otits externa	2.9 % of 202 cases caused by *S. caprae*
Devrise *et al.* and Barelli *et al.* 1999 [2, 14]	Scientific review articles	Nosocomial infections, varied	3 of 53 cases were caused by *S. caprae*
Benedetti *et al.* 2008 [5]	Case report	CSF infection	47-year-old female with lumbar-sacral s/p spinal analgesia pump implantation, required device removal and 6 weeks IV antibiotics
Kato *et al.* 2010 [8]	Case report	Bacteremia	Neutropenic patient after induction chemotherapy for ac, associated with central line
Shin *et al.* 2011 [15]	Case report	Peritonitis	3 patients receiving peritoneal dialysis
Henry *et al.* 2014 [7]	Case report	Endophthalmitis	24-year-old healthy female after surgery for vitreous floaters

There are two major studies looking at the incidence of *S. caprae* bone and joint infections. The first, by d’Ersu *et al.* [6], was a retrospective study done at Nantes University looking at data between 2004 and 2012. They identified 13 patients with *S. caprae* bone and joint infections, and in this study four patients had infection of their native bone: two individuals with diabetic foot infections, one with recurrent osteomyelitis and one with chronic osteitis [6].

The second study was published by Seng *et al.* [4] and looked at two major hospitals in France between 2006 and 2012, the University of Marseille and the University of Nimes. The University of Marseille had 16 bone and joint infections from 2006 to 2012 caused by *S. caprae* and the University of Nimes had nine bone and joint infections from 2007 to 2012 caused by *S. caprae*. In this study, only one patient had infection of a vertebra, and only three patients had no orthopaedic devices present at the time of infection. Most patients required more than two antibiotics with surgical debridement and removal of the orthopaedic device for complete clearance of infection [4].

Interestingly, a study published in 2014 looking at small colony variations in bone and joint infections found that in 76 human samples only two grew *S. caprae/Staphylococcus capitis* and that distinguishing between the two can be difficult when using the 16S rRNA gene, which is considered the gold standard [11, 12]. Furthermore, in the study of d’Ersu *et al*. from 2016 they found that a small number of isolates were misidentified by a commercial identification system. MALDI-TOF MS was found to be far superior in identification rates of *S. caprae* [6].

For our individual patient, both failure of antibiotic therapy and source control likely contributed to his ultimate clinical outcome. In the available literature it is clear that without removal of infected bone or devices, antibiotic clearance of the infection did not occur. Unfortunately, given his comorbidities and rapid clinical decline, our patient was not a surgical candidate. Additionally, production of toxins by this strain of *S. caprae* was not tested.

In conclusion, *S. caprae* is still an understudied organism of human infection, and it has been determined to carry the *mecA* gene conferring methicillin resistance making it an important pathogen to be identified early on, especially in patients with significant comorbidities.
